# P-glycoprotein mediated efflux limits substrate and drug uptake in a preclinical brain metastases of breast cancer model

**DOI:** 10.3389/fphar.2013.00136

**Published:** 2013-11-04

**Authors:** Chris E. Adkins, Rajendar K. Mittapalli, Vamshi K. Manda, Mohamed I. Nounou, Afroz S. Mohammad, Tori B. Terrell, Kaci A. Bohn, Celik Yasemin, Tiffany R. Grothe, Julie A. Lockman, Paul R. Lockman

**Affiliations:** ^1^Department of Pharmaceutical Sciences, School of Pharmacy, Texas Tech University Health Sciences CenterAmarillo, TX, USA; ^2^Department of Life, Earth and Environmental Sciences, West Texas A&M UniversityCanyon, TX, USA

**Keywords:** drug resistance, chemotherapy, autoradiography, fluorescence microscopy, tumor

## Abstract

The blood–brain barrier (BBB) is a specialized vascular interface that restricts the entry of many compounds into brain. This is accomplished through the sealing of vascular endothelial cells together with tight junction proteins to prevent paracellular diffusion. In addition, the BBB has a high degree of expression of numerous efflux transporters which actively extrude compounds back into blood. However, when a metastatic lesion develops in brain the vasculature is typically compromised with increases in passive permeability (blood-tumor barrier; BTB). What is not well documented is to what degree active efflux retains function at the BTB despite the changes observed in passive permeability. In addition, there have been previous reports documenting both increased and decreased expression of P-glycoprotein (P-gp) in lesion vasculature. Herein, we simultaneously administer a passive diffusion marker (^14^C-AIB) and a tracer subject to P-gp efflux (rhodamine 123) into a murine preclinical model of brain metastases of breast cancer. We observed that the metastatic lesions had similar expression (*p* > 0.05; *n* = 756–1214 vessels evaluated) at the BBB and the BTB. Moreover, tissue distribution of R123 was not significantly (*p* > 0.05) different between normal brain and the metastatic lesion. It is possible that the similar expression of P-gp on the BBB and the BTB contribute to this phenomenon. Additionally we observed P-gp expression at the metastatic cancer cells adjacent to the vasculature which may also contribute to reduced R123 uptake into the lesion. The data suggest that despite the disrupted integrity of the BTB, efflux mechanisms appear to be intact, and may be functionally comparable to the normal BBB. The BTB is a significant hurdle to delivering drugs to brain metastasis.

## INTRODUCTION

The successful treatment of central nervous system (CNS) tumors and metastases using chemotherapy depends on the ability of therapeutic concentrations of drug to cross the blood–brain barrier (BBB). More than 98% of potential CNS active anticancer drugs fail in preclinical work and or clinical trials because of inadequate BBB penetration (Pardridge, 2007). Clinically this results in many anticancer agents failing to substantially reduce tumor burden and or significantly prolong survival (Deeken and Loscher, 2007).

The microvasculature of the brain is a unique anatomical structure which serves as a homeostatic and regulatory barrier between the blood and the brain parenchyma (Hawkins and Davis, 2005). Specifically, endothelial cells that line the blood vessels of the brain capillaries are fused together by numerous tight junction protein complexes, which restrict blood components from passively diffusing between the cell margins to gain entry into brain. The tight junction protein complexes consist of a number of proteins such as zonula occludins, junctional adhesion molecules, and claudins which function as a unit to seal the endothelia margins. Further the outside of the brain capillary is surrounded by astrocytic foot processes and pericytes that also contribute to the restriction of paracellular diffusion (Abbott et al., 2010).

Further restricting the brain entry of a large number of drugs and drug classes are efflux transporters at the BBB. Efflux transporters are richly expressed in the brain vasculature and have been shown to restrict the accumulation of antiepliptics, antidepressants, and antipsychotics (Schinkel et al., 1995; Loscher and Potschka, 2005). Multiple efflux transporters at the BBB act to actively extrude or prevent drug accumulation into brain, these include P-glycoprotein (P-gp; ABCB1) (Schinkel et al., 1996), breast cancer resistant protein (BCRP; ABCG2; Polli et al., 2009), multidrug resistance associated proteins (MRP; ABCC1-6; Breedveld et al., 2005), and organic anion transporters (OATs; Hagenbuch and Meier, 2004).

The net effect of the anatomical and molecular features of the BBB is that to a large degree it restricts drug movement from blood into brain. But some drugs are able to penetrate the BBB. Drug and/or solute permeation across the BBB is mostly limited to low molecular weight lipid-soluble molecules. Molecules that are large (typically >700 Da), protein bound or are hydrophilic will have difficulty crossing the BBB and accumulating in brain to a sufficient degree (Lipinski et al., 2001).

However, the vasculature within a brain tumor is different from the normal BBB. Previously it has been shown that the blood-tumor barrier (BTB) vasculature has disrupted integrity compared to the intact BBB. This disruption can allow small molecule to accumulate into lesions up to 30–100-fold more than the accumulation of the molecule in normal brain (Lockman et al., 2010; Taskar et al., 2012). While the degree of breakdown at the BTB does correlate with increases in drug uptake it is not clearly defined whether efflux pumps continue to limit drug uptake into metastatic lesions (Gallo et al., 2003). It has been previously shown that the BTB expresses P-gp (Cordon-Cardo et al., 1990); however, the expression of P-gp may be variable among different tumors types (Henson et al., 1992). In addition to P-gp expression at the BTB, many cancers have been shown to express functional P-gp *in vivo *which may restrict the cellular accumulation of chemotherapuetics.

Herein we set out to determine the expression and function of P-gp in a preclinical model of brain metastases of breast cancer using quantitative fluorescence microscopy and autoradiography. We observed that P-gp is expressed at the BTB in brain metastases at nearly similar levels to the BBB. In addition, P-gp is highly functional in limiting the lesion accumulation of the P-gp substrate, Rhodamine 123 (R123) despite significant passive permeability increases.

## MATERIALS AND METHODS

### CHEMICALS

R123 was purchased from Molecular Probes Invitrogen (Eugene, OR, USA). Verapamil was purchased from Sigma (St. Louis, MO, USA). Cyclosporine A was purchased from Tocris Biochemicals (St. Louis, MO, USA). ^14^C-labeled aminoisobutyric acid (AIB) was purchased from American Radiolabelled Chemicals (St. Louis, MO, USA). All other chemicals used were of analytical grade and were used as supplied.

### ANIMALS

Female NuNu mice (~24 g; 8 weeks of age) were purchased from Charles River Laboratories (Kingston, NY, USA) and were used for all the perfusion experiments done in this study. All studies were approved by the Animal Care and Use Committee and were performed in accordance with the NIH Guidelines for the Care and Use of Laboratory Animals.

### *IN SITU* MOUSE HEART PERFUSION TECHNIQUE

The *in situ* mouse heart perfusion technique was utilized to evaluate brain uptake of R123 (Takasato et al., 1984; Lockman et al., 2003a) Mice were anesthetized with ketamine/xylazine (100 and 8 mg/kg, respectively) and the heart exposed. Body temperature was monitored and maintained at 37°C using a heating pad attached to a feedback device (YSI Indicating Controller, Yellow Springs, OH, USA). Prior to insertion of the cannula, the right cardiac atrium was cut to prevent venous return. Cannulation of the left cardiac ventricle was done using butterfly syringe (28G) attached to a perfusion apparatus. Perfusion fluid was pumped into the left cardiac ventricle by a cannula at a constant rate of 2.5 mL/min (Dagenais et al., 2000) using a Harvard Model 944 dual channel pump (Harvard Apparatus, South Natick, MA).

The perfusion fluid consisted of HCO_3_ buffered physiological saline, containing 128 mM NaCl, 24 mM NaHCO_3_, 4.2 mM KCl, 2.4 mM NaH_2_PO_4_, 1.5 mM CaCl_2_, 0.9 mM MgSO_4_, and 9 mM glucose (pH ~7.35; [Na] = 154.4 mM). All solutions were filtered, oxygenated, warmed to 37° C, and adjusted to pH 7.35 prior to perfusion. To determine initial brain uptake of R123, perfusion fluid containing R123 (50 μg/mL) was infused into the systemic circulation for 30–120 s. At the end of each experiment, mice were sacrificed, and the brain was rapidly removed (<60 s) from the skull. The brain was flash frozen in isopentane (-65°C). Concentration of the fluorophore (R123) in brain was determined using fluorescent microscopy and regional permeability was expressed by the unidirectional transfer constants, K_in_ (mL/s/g) derived from Eq. 1.

### QUANTIFICATION OF R123 USING FLUORESCENCE MICROSCOPY

Fluorescence was observed with an Olympus MVX10 stereomicroscope (objective: 2×, NA 0.5) with an optical zoom range from 0.63 to 12.6. The excitation and emission of R123 was obtained using a GFP filter (excitation/band pass filter of 470/40, emission/band pass filter of 525/50 and dichromatic mirror at 495 nm; Chroma Technology, Bellow Falls, VT, USA). Tissue sections of 20 μm were obtained at -23°C using a cryotome (Leica CM3050S, Leica Microsystems, Buffalo Grove, IL, USA), mounted on charged glass slides, and kept at -23°C. Data were analyzed using quantitative fluorescence microscopy and all images were obtained with 15 ms exposures, though a 2.0 objective at 4× magnification (Olympus MVX10) with a monochromatic cooled CCD scientific camera (Retiga 4000R, QImaging, Surrey, BC, Canada). Slidebook^®^ 5 software (Intelligent Imaging Innovations, Denver, CO, USA) was utilized to determine sum intensity per gram of brain which then converted into concentration of dye per gram of brain using the brain homogenate standards. The voxel by voxel sum intensity of fluorescence for brain homogenate samples was obtained with the 2× objective. The optical zoom range was maintained at 4× for a total optical magnification of 8×. The sum intensity per gram of brain homogenate was obtained using a set exposure time of 15 ms with camera gain settings of 615. The total fluorescence intensity signal for each concentration was then plotted as a function of grams of brain which was calculated using the area in microns squared multiplied by the thickness of the brain sample (20 μm) to obtain a total brain volume that was analyzed. The brain volume (μm^3^) was multiplied by the density of brain tissue (1.04 g/cm^3^) as similarly reported by (Tengvar et al., 1983) to obtain a weight of brain tissue that was analyzed.

### PREPARATION OF BRAIN STANDARDS

To calculate the concentration of the R123 in brain, standard curves were generated in rat brain homogenates. Briefly, 100 μL of standard solution of the dye was added to each of 500 mg of the brain and homogenized. The homogenized mass was flash frozen in isopentane (-80°C) and sliced into 20 μm sections using a cryostat -23°C and mounted onto glass, superfrost slides. The slices were analyzed using quantitative fluorescence microscopy and the sum intensity per gram of brain homogenate was plotted against concentration of the dye.

### KINETIC ANALYSIS

Unidirectional uptake transfer constants (*K*_in_) were calculated from the following relationship to the linear portion of the uptake curve:

(1) $$Q^{*}/C^{*}=K_{i\,n}\,T+V_{0}$$

where Q* is the quantity of fluorophore (R123) in brain (μg/g) at the end of perfusion, C* is the perfusion fluid concentration of fluorophore (μg/mL), *T* is the perfusion time (s) and *V*_0_ is the extrapolated intercept (*T* = 0 s; “vascular volume” in mL/g). After determination of a perfusion time that allowed adequate amount of fluorescent marker to pass into brain and yet remained in the linear uptake zone, *K*_in_ was determined in single time-point experiments as:

(2) $$K_{i\,n}=\left[Q^{*}-V_{0}\,C^{*}\right]/C^{*}\,T]$$

(Takasato et al., 1984; Smith and Takasato, 1986).

### ANTIBODY STAINING

Tissues were rehydrated in PBS and then fixed in 4% paraformaldehyde (PFA) for P-gp (Abcam, Cambridge, MA), cytokeratin (Abcam) and CD31 (BD Pharmingen, San Jose, CA), ice-cold methanol for ABCB1 (Santa Cruz Biotechnology), CD31 (BD Pharmingen). After three PBS washings (5 min), slides were blocked with 4% goat serum and 0.2% Triton-X 100 (1 h). After blocking, primary antibodies were added, followed by overnight incubation at 4°C. The next day, the slides were washed and secondary antibodies and DAPI (1 mg/mL) were added (1 h). Slides were again washed, DAKO mounting medium was added, and coverslips were applied.

## RESULTS

**FIGURE 1:**
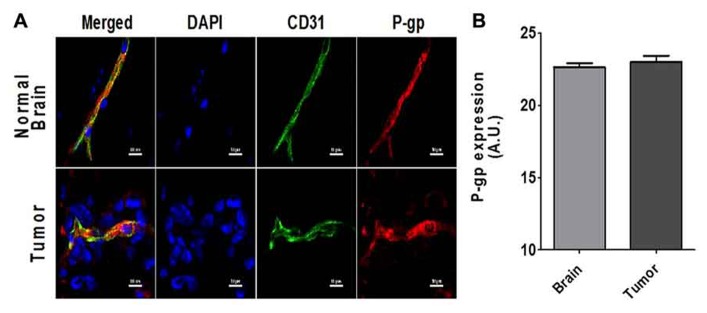
**(A)** Representative images of the co-localized expression of P-gp (red) in a capillary in normal brain (top row) and a blood vessel in a 231Br brain metastasis (bottom row) are shown. Endothelial nuclei as well as the nuclei of the 231Br lesions are shown in blue (DAPI). Blood vessels (CD-31 expression) in both sections are shown in green (Alexa Fluor 488). P-gp expression is shown in red (Alexa Fluor 594). **(B)** The bar graph shows the relative P-gp expression per vessel as defined by CD31 stained regions. Mean + SEM; BTB; *n* = 756 vessels and BBB *n* = 1214 vessels).

To determine if P-gp expression is present in the vasculature of brain metastases, we analyzed the brains of tumor bearing mice using immunofluorescence staining for both P-gp and the vascular marker CD31 to quantify the amount of colocalization (**Figure 1**). There was significant expression of P-gp at the BBB and BTB (**Figure 1B**). Overall there was no difference between the fluorescent intensity of P-gp staining in the CD-31 defined regions in tumor vasculature (22.9 + 0.4 A.U.; *n* = 756 vessels) and in the normal brain vasculature (22.6 + 0.3 A.U.; *n* = 1214). In addition, there was positive P-gp staining that did not co-localize to the vasculature, but surrounded metastasis cells suggesting that P-gp may also be present on the metastatic cancer cells.

**FIGURE 2:**
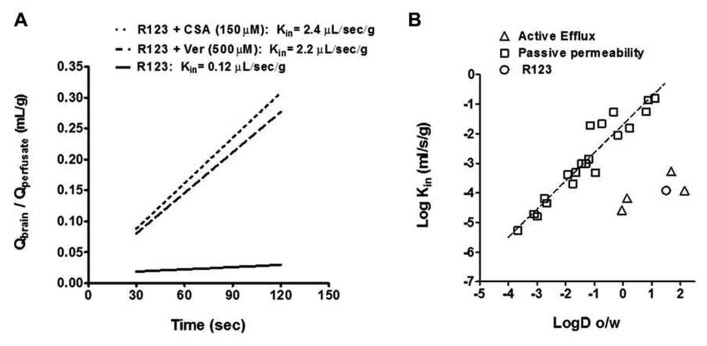
**(A)** The *K*_in_ of R123 in the presence of the known P-gp inhibitors, cyclosporin A and verapamil, increases brain distribution (as reported by the *K*_in_) by >10-fold. All data represent mean ± S.E.M for total brain; *n* = 3–5 for all groups. Statistics: one-way ANOVA; Dunnett’s. **(B) **The relationship between LogD (octanol/water coefficient; pH = 7.4) and observed *K*_in_ is used to profile a molecule or drug’s mechanism of distribution into brain. Compounds that are known to cross the BBB via passive diffusion are plotted using gray squares and those subject to efflux are plotted with gray triangles. R123 (open circle) falls below ~3 log units the line of identity for passive permeability indicating it may be subject to efflux.

We measured P-gp function by the time dependent accumulation of the fluorescence P-gp substrate R123 according to previous methodology (Mittapalli et al., 2013). Using fluorescent brain standards we determined the blood to brain unidirectional transfer coefficient (*K*_in_) of R123 in normal brain and in metastatic lesions by calculating the concentration of R123 divided by the concentration in the perfusate and plotted this over time (30–120 s; **Figure 2A**). We then applied a previously calculated correction to the vascular volume by perfusion of non-permeable [^14^C]-sucrose and measuring its vascular space (0.015 ± 0.002 mL/g). We observed that the uptake of R123 was linear within the perfusion time with a *K*_in_ of 0.12 ± 0.03 μL/s/g. To determine if we could inhibit P-gp mediated efflux of R123, we added P-gp inhibitors verapamil and cyclosporine A (Choi and Li, 2005; Breedveld et al., 2006; Baumert and Hilgeroth, 2009) at various concentrations to the R123 perfusate in separate experiments (**Figure 2A**). Upon co-perfusion of R123 and each inhibitor, there was an increase in R123 permeability; Cyclosporin A (2.4 ± 0.5 μL/s/g); and Verapamil (2.2 ± 0.2 μL/s/g)] indicating that R123 uptake into brain is limited by the efflux function of P-gp at the BBB.

We then plotted R123’s LogD (octanol/water coefficient; pH = 7.4) and observed *K*_in_ in comparison to known passive permeability compounds (Begley, 1996) and efflux substrates (Summerfield et al., 2007; **Figure 2B**). Molecules and drugs that passively diffuse into brain exhibit a linear relationship between their LogD (octanol/water coefficient) and their observed Log *K*_in_ while molecules which are subject to efflux will exhibit observed Log *K*_in_ values well below the value predicted by its LogD (**Figure 2B**). R123 has a LogD of 1.51 (Forster et al., 2012) and Log *K*_in_ of -3.93 (calculated from observed *K*_in_; **Figure 2A**) which places R123 several orders of magnitude below a passively diffusing molecule’s profile which supports the evidence of R213’s restriction from brain via an efflux transporter.

**FIGURE 3:**
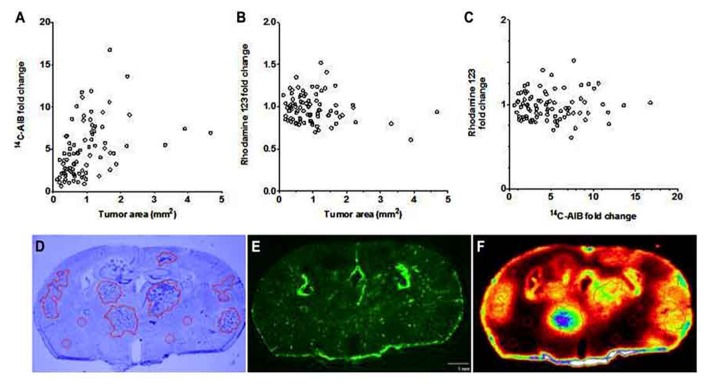
**The BTB is variably compromised for compounds entering via passive diffusion but retains P-gp mediated efflux.** The passive permeability marker ^14^C-AIB fold change in brain metastases did not correlate (*r*^2^ = 0.167) with metastasis size (mm^2^) **(A)**. R123 fold change in brain metastases did not correlate (*r*^2^ = 0.033) with metastasis size **(B)**. There was no observed relationship (*r*^2^ = 0.0009) between the fold increase in brain metastases of ^14^C-AIB (passive permeability marker) and R123 (P-gp substrate) **(C)**. One representative brain slice showing metastases location (**D**, cresyl violet), R123 fluorescence distribution (**E**, fluorescence microscopy), and ^14^C-AIB brain uptake (**F**, quantitative autoradiography).

**FIGURE 4:**
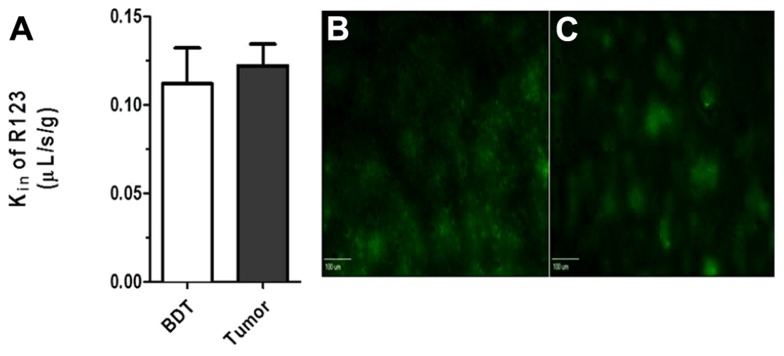
**(A)** No difference (*p* > 0. 05; student *t*-test, *n* = 3–5) was seen between R123 blood to brain transfer constant *K*_in_ between values measured in normal brain (*K*_in_ = 0.11 + 0.01 μL/s/g) and metastases (*K*_in_ = 0.12 ± 0.02 μL/s/g). Representative R123 fluorescence images in normal brain **(B)** and within a metastasis **(C)** (scale bar = 100 μm).

To determine BTB passive permeability and whether P-gp influences R123 uptake into brain metastases of breast cancer, tumor-bearing mice were injected with ^14^C-AIB (passive permeability tracer) which was allowed to circulate for 10 min before a 2 min R123 perfusion, which was followed by sacrifice (**Figure 3**). Autoradiography analysis of the brains revealed elevated permeability to ^14^C-AIB (~4.9-fold increase). The passive permeability marker tracer’s uptake did not correlate (*r*^2^ = 0.17 for AIB) with metastases size (**Figure 3A**). R123 uptake, however, was not different from that of normal brain on average (~0.98-fold change) in metastatic lesions and had no correlation (*r*^2^ = 0.033) to metastasis size (**Figure 3B**). R123 permeability did not correlate passive permeability changes as measured by ^14^C-AIB (*r*^2^ = 0.0008) (**Figure 3C**) accumulation suggesting that R123 remains restricted from the brain parenchyma via P-gp mediated efflux. The observed R123 *K*_in_ value for normal brain (BDT; brain distant to tumor) regions of metastases bearing mice (*K*_in_ = 0.11 ± 0.06 × 10^-^^1^μL/s/g) (**Figures 4A–C**) was consistent with previous *K*_in_ measurements in tumor-free mice (**Figure 1B**). And, the *K*_in_ of R123 in the BTB (within metastases) was 0.12 ± 0.003 μL/s/g which was not different than that of normal brain (*p* > 0.05).

## DISCUSSION AND CONCLUSION

In the current study, we present data suggesting P-gp retains its efflux function at the BTB despite a disruption in the integrity of the BBB induced by the presence of a metastatic lesion. Of notable methodology, to the best of our knowledge we are the first to combine quantitative fluorescence microscopy to measure R123 P-gp mediated efflux and quantitative autoradiography to measure changes in BTB passive permeability (^14^C-AIB) in the same brain slice. This method is able to directly shed light on two independent processes occurring at the BTB.

The utilization of R123 to evaluate P-gp function is well established (Hegmann et al., 1992). However, there is less evidence regarding R123’s affinity and efflux transport to other transporters that contribute to drug restriction to brain. R123 has been reported to be subject to transport by BCRP (Doyle et al., 1998), and OCT 1 & 2 (Jouan et al., 2012), and MRP2 (Munic et al., 2011). Though studies using specific transporter inhibitors at correct concentrations show P-gp primarily transports R123 and restricts accumulation into brain (Wang et al., 1995). Moreover, the magnitude of R123 efflux by P-gp is greater than that of BCRP and MRP1 (Chopra, 2004) and therefore should represent the major pathway of active efflux transport at the BBB and BTB.

Due to the difficulty in performing the *in situ* brain perfusion in mice, we modified the *in situ* brain perfusion to a cardiac perfusion method in female Nu/Nu mice bearing brain metastases of breast cancer to characterize P-gp function *in vivo*. This method has similar advantages to the *in situ* brain perfusion method in that we may control aspects of the perfusion to determine both influx and efflux kinetics, transporter inhibition coefficients, and BTB or BBB permeability (Smith and Allen, 2003). This control helps determine accurate apparent permeability coefficients (Lockman et al., 2005a), the degree to what a substrate is efflux back into blood (Lockman et al., 2003b), inhibition constants for transporters (Lockman et al., 2001) and a direct measurement of BBB and BTB integrity (Lockman et al., 2003a, 2004, 2005b)

Using the cardiac perfusion method, R123 accumulated in brain linearly over 2 min of perfusion time. Our observed blood to brain transfer constant (*K*_in_) was ~10-fold less than what would be the calculated *K*_in_ based on values of similar molecules in terms of their octanol/water coefficient and molecular weight. The lower observed *K*_in_, is typically seen when the compound is actively extruded by the BBB back into blood (Begley, 1996). Further confirmation that R123 is extruded by an efflux mechanism at the BBB was suggested by the significantly increased uptake of R123 from blood to brain after the addition of verapamil or cyclosporine A to the perfusate (Mittapalli et al., 2013).

Of importance to this study, the simultaneous administration of a passive permeability marker and a tracer subject to P-gp mediated efflux allowed us to measure BTB integrity and functional efflux. Both parameters have been shown to significantly impact drug uptake into metastases (Lockman et al., 2010) but have not been simultaneously measured directly in metastatic lesions. Our initial hypothesis prior to the experiment was that since we have seen increased permeability at the BTB in metastases (Lockman et al., 2010), we would also see a similar increase in R123 distribution into the lesion. However we did not observe R123 accumulation within metastases.

There are two possible explanations that may provide insight to the lack of increased R123 permeability in the lesion. First, it is known that P-gp is expressed in the vasculature of human brain tumors and metastases (Guo et al., 2010). Although, P-gp expression at the BTB has been shown to be variable among different types of tumors within the CNS (Cordon-Cardo et al., 1990; Toth et al., 1996; Tews et al., 2000) as well as different between separate intracranial metastases (Demeule et al., 2001; Lockman et al., 2010). We observed some variability of P-gp expression in the vessels of our metastases, but overall P-gp expression was not significantly different in the over 2,000 vessels we analyzed between the BTB and the BBB. Accordingly, this may be a reason why there was little overall difference in tissue accumulation of R123 between the two tissue types. Another possible explanation is that we observed tumor cells directly adjacent or proximal to the vasculature also express P-gp, which may also contribute to the restriction of R123 in the lesions. Overall, the pattern of distribution for each tracer suggests that the BTB is disrupted yet its efflux transport mechanisms are intact and can limit brain and or tumor uptake of P-gp substrates.

This work does have translational value to human drug distribution to brain. The expression of BCRP at human BBB is ~2 fold higher as compared to the expression levels at mouse BBB. The P-gp expression is 3 fold higher at mouse BBB as compared to the expression levels at human BBB. So BCRP still plays a major role at human BBB (Hoshi et al., 2013) suggesting P-gp plays a major functional role in the human BBB. While some studies have supported little efflux contribution for various anti-cancer drug to brain (Agarwal et al., 2011), others have demonstrated P-gp at the BBB and BTB restricts the uptake of many anti-cancer agents; such as paclitaxel, docetaxel, vemurafenib, erlotinib, axitinib, and tamoxifen (Gallo et al., 2003; Kemper et al., 2004; Wang et al., 2010; Iusuf et al., 2011; Poller et al., 2011; Mittapalli et al., 2012; Taskar et al., 2012; Agarwal et al., 2013). Attempts to modify P-gp using inhibitors have shown promise in preclinical settings (Kemper et al., 2004; Mittapalli et al., 2012; Agarwal et al., 2013).

Although we, and others, have observed variably elevated accumulations of small molecules across the BTB in brain metastases, the data herein provide evidence that P-gp retains much of its residual function. Thus, BTB function in this preclinical model may be viewed as only partially compromised and retains significant ability to impede uptake of therapeutic compounds. Given the large list of drugs, particularly anticancer agents such as paclitaxel and doxorubicin, which are subject to P-gp mediated efflux, the clinical impact of this retained function suggests the BTB remains a significant barrier in delivering chemotherapeutics into metastatic lesions.

## AUTHORS’ CONTRIBUTIONS

1. Concept and design – Chris E. Adkins, Rajendar K. Mittapalli, Vamshi K. Manda, Paul R. Lockman
2. Development of methodology Chris E. Adkins, Rajendar K. Mittapalli, Vamshi K. Manda, Kaci A. Bohn, Paul R. Lockman
3. Acquisition of data (provided animals, acquired and managed patients, provided facilities, etc) Chris E. Adkins, Rajendar K. Mittapalli, Vamshi K. Manda, Tori B. Terrell, Kaci A. Bohn, Celik Yasemin, Tiffany R. Grothe, Julie A. Lockman, Paul R. Lockman
4. Analysis and interpretation of data (e.g., statistical analysis, biostatistics, computational analysis) Chris E. Adkins, Rajendar K. Mittapalli, Vamshi K. Manda, Mohamed I. Nounou, Afroz S. Mohammad, Tori B. Terrell, Julie A. Lockman, Paul R. Lockman
5. Writing, review, and/or revision of the manuscript Chris E. Adkins, Mohamed I. Nounou, Afroz S. Mohammad, Tori B. Terrell, Paul R. Lockman
6. Study supervision Paul R. Lockman

## Conflict of Interest Statement

The authors declare that the research was conducted in the absence of any commercial or financial relationships that could be construed as a potential conflict of interest.
